# Clinical warning signs preceding severe suicide attempts requiring pediatric intensive care in children and adolescents

**DOI:** 10.3389/fpsyt.2026.1881780

**Published:** 2026-07-27

**Authors:** Merve Boyraz, Servet Yüce, Gülsüm Feyza Kızıltan, Hazal Ceren Turgut, Osman Faruk Bayramlar, Edin Botan

**Affiliations:** 1 Department of Pediatrics, Van Research and Training Hospital, Health Sciences University, Van, Türkiye; 2 Department of Public Health, İstanbul Provincial Health Directorate, İstanbul, Türkiye; 3 Department of Obstetrics and Gynecology, Private Lokman Hekim Hayat Hospital, Van, Türkiye; 4 Department of Pediatrics, Division of Pediatric Critical Care, Van Research and Training Hospital, Health Sciences University, Van, Türkiye; 5 Haver Pharma, Istanbul, Türkiye

**Keywords:** adolescent mental health, childhood suicide, pediatric intensive care unit, suicide attempt, suicide prevention, warning signs

## Abstract

**Background:**

Childhood suicide attempts requiring pediatric intensive care unit (PICU) admission represent some of the most severe forms of self-harm and are associated with substantial morbidity and mortality. This study aimed to identify potential psychosocial factors and clinical warning signs preceding severe suicide attempts in children and adolescents.

**Methods:**

This retrospective study was conducted in the PICU of Van Regional Training and Research Hospital between January 2017 and January 2025. Children aged 8–18 years admitted following intentional self-harm or suicide attempts were eligible. Survivors participated in structured face-to-face interviews. For deceased patients, information was obtained from at least three first-degree relatives and corroborated using medical records and reports from treating physicians.

**Results:**

During the study period, 256 individual patients accounted for 289 suicide-related PICU admissions, of whom 116 met the inclusion criteria. The cohort included 81 females (69.8%) and 35 males (30.2%), with a mean age of 15.5 ± 2.1 years. Patients were classified according to the presence of documented healthcare encounters within the preceding year: Group 1 (80/116, 69.0%) had at least one prior healthcare contact for potential warning signs, whereas Group 2 (36/116, 31.0%) had none. Group 2 patients were younger, more frequently aged 8–13 years, and experienced significantly higher morbidity and mortality. Mortality was significantly higher among boys aged 8–13 years. The highest mortality rates were observed among patients with gender identity-related distress (3/3) and pregnant adolescents reporting violations of sexual consent (3/4), although these findings were based on very small subgroups.

**Conclusions:**

Nearly 70% of children who attempted suicide had prior healthcare encounters for potentially recognizable warning signs, including psychosomatic complaints, humiliation or bullying, exposure to violence, and gynecological presentations. These findings highlight opportunities for earlier recognition of psychosocial distress during routine clinical encounters. Given the retrospective single-center design and absence of a non-suicidal comparison group, the results should be considered exploratory and hypothesis-generating rather than predictive.

## Background

Suicide is the second leading cause of death among children and adolescents and represents a major global public health concern (1). In the United States, suicide rates among pediatric age groups have increased substantially over recent decades, with many serious attempts resulting in admission to pediatric intensive care units (PICUs) (2). This persistent rise in childhood and adolescent suicide has prompted increasing recognition of suicide prevention as a public health priority (1, 3).

Numerous psychosocial and environmental factors have been associated with suicidal thoughts and behaviors in children and adolescents. These include appearance-related distress, experiences related to sexual orientation or gender identity, physical, sexual, and emotional abuse, neglect, domestic violence, bullying, discrimination, humiliation, family conflict, and social isolation (1). Suicide attempts requiring PICU admission are typically among the most severe and potentially lethal forms of self-harm, placing these patients within a particularly high-risk population. Consequently, increasing attention has been directed toward identifying potentially recognizable warning signs that may precede severe suicide attempts (2, 4, 5).

Importantly, the distribution and expression of these factors vary considerably according to sociocultural, economic, ethnic, and geographic contexts. Even within the same country, substantial differences may exist between regions, cities, and communities (1, 2, 6, 7). Understanding local patterns of vulnerability may facilitate earlier recognition of at-risk children and adolescents, strengthen preventive interventions, and support timely referral to mental health services when indicated (1, 2, 4, 8).

Data from low- and middle-income countries remain relatively limited, particularly regarding severe suicide attempts requiring PICU admission (6, 9). Furthermore, recent data from Türkiye have demonstrated increasing rates of suicidal ideation and suicide attempts among adolescents during and after the COVID-19 pandemic. In a tertiary adolescent medicine center, suicidal ideation increased 1.4-fold and suicide attempts increased 1.5-fold compared with the pre-pandemic period, highlighting the importance of routine psychosocial assessment and suicide risk screening in healthcare settings (10). Accordingly, the present study aimed to identify potentially recognizable clinical warning signs and psychosocial factors preceding serious suicide attempts among children and adolescents admitted to a PICU in Eastern Türkiye. Rather than establishing validated predictors of suicide, our goal was to describe potentially important warning signs that may help clinicians across specialties recognize vulnerable patients and facilitate earlier intervention (2, 4, 5).

## Methods

### Study design and population

This retrospective study evaluated children and adolescents aged 8–18 years who were admitted to the PICU following intentional self-harm or suicide attempts between January 2017 and January 2025.

### Study design and data collection

Surviving patients participated in structured face-to-face interviews exploring the circumstances preceding their suicide attempt. Interview questions included: “Why did you want to end your life?”, “What made you unhappy?”, and “What was the biggest problem you remember facing at that time?” Additional questions were asked when relevant, including inquiries regarding marriage, pregnancy, and romantic relationships.

For deceased patients, information was obtained through interviews with at least three first-degree relatives (parents or siblings) or other close family members who had substantial involvement in the child’s life. Interviews with family members of deceased patients were conducted by two investigators (MB and SY), both physicians experienced in pediatric patient interviews, between 3 and 12 months after the suicide event whenever possible. Information was cross-checked for consistency and corroborated using available medical records, forensic reports, discharge summaries, and, when available, prosecutor’s reports. In cases of conflicting accounts, only information consistently reported by multiple sources and supported by available documentation was included in the analysis.

Because information regarding deceased patients was obtained retrospectively, the possibility of recall and reporting bias was considered during data interpretation.

### Study setting

The study was conducted in Van, a metropolitan city in Eastern Türkiye with a population of approximately 1.2 million. Van Regional Training and Research Hospital is the major tertiary referral center for the region, serving eight surrounding provinces and approximately 50 districts. The hospital provides healthcare for an estimated catchment population of nearly 10 million people.

The PICU is a tertiary-level unit with 29 beds and offers advanced therapies including dialysis and extracorporeal membrane oxygenation (ECMO). Approximately 1,500 patients are admitted annually.

During the study period, 256 individual patients accounted for 289 suicide-related PICU admissions. Because some patients experienced multiple admissions, the total number of admissions exceeded the number of individual patients. After application of the predefined inclusion and exclusion criteria, 116 patients were included in the final analysis.

### Inclusion criteria

1. Children and adolescents aged 8–18 years admitted to the PICU following a first suicide attempt during the study period.
2. Survivors who agreed to participate in face-to-face interviews.
3. Deceased patients for whom at least three family members agreed to participate, provided consistent information, and whose accounts could be corroborated using available documentation.

### Exclusion criteria

1. Survivors who declined participation or provided incomplete or inconsistent information.
2. Deceased patients for whom fewer than three family members could be interviewed.
3. Patients with suspected intoxication unrelated to self-harm or with acute neurological infections (e.g., meningitis or encephalitis).
4. Cases suggestive of homicide or cases under active legal investigation in which family interviews were prohibited.
5. Patients with previously established major psychiatric disorders (e.g., schizophrenia spectrum disorders, bipolar disorder, or severe anxiety disorders). These patients were excluded because the primary objective was to identify potentially overlooked warning signs and psychosocial stressors among children without a known psychiatric diagnosis. Nevertheless, this exclusion may have introduced selection bias and may limit the generalizability of the findings to the broader population of adolescents at risk for suicidal behavior.
6. Patients whose contact information could not be obtained.

### Variables and outcomes

Collected variables included demographic characteristics, suicide method, clinical outcomes, and psychosocial factors identified through interviews and medical record review.

Hospital encounters occurring within one year before the suicide attempt were reviewed to identify potential warning signs that may have been recognizable to healthcare professionals. Because the study was limited to records available within our institution, healthcare encounters occurring at other hospitals could not be systematically assessed.

Potential warning signs were retrospectively categorized into five predefined groups:

1. Exposure to violence,
2. Humiliation or bullying-related presentations,
3. Recurrent psychosomatic complaints,
4. Premature discharge against medical advice,
5. Gynecology-related presentations.

Patients could be assigned to more than one category when multiple warning signs were identified.

For patients with multiple suicide-related admissions, only the first eligible admission was included in the analysis.

Patients were stratified into two age groups: 8–13 years and >13–18 years, representing younger children/early adolescents and older adolescents, respectively (11).

### Statistical analysis

All statistical analyses were performed using IBM SPSS Statistics for Mac, Version 30.0 (IBM Corp., Armonk, NY, USA). The distribution of continuous variables was evaluated using the Shapiro–Wilk test and visual inspection of histograms and Q–Q plots. Normally distributed variables are presented as mean ± standard deviation (SD), whereas non-normally distributed variables are presented as median (minimum–maximum). Comparisons between independent groups were performed using the independent-samples t-test or Mann–Whitney U test, as appropriate. Categorical variables are reported as frequencies and percentages and were compared using the chi-square test or Fisher’s exact test when expected cell counts were less than five. Because only 13 mortality events occurred in the study cohort, multivariable logistic regression was not performed due to the high risk of model overfitting and unstable effect estimates. Consequently, associations between patient characteristics and outcomes were evaluated using univariate analyses only. All statistical tests were two-sided, and p-values <0.05 were considered statistically significant.

## Results

### Study population and sociodemographic characteristics

**Figure 1:**
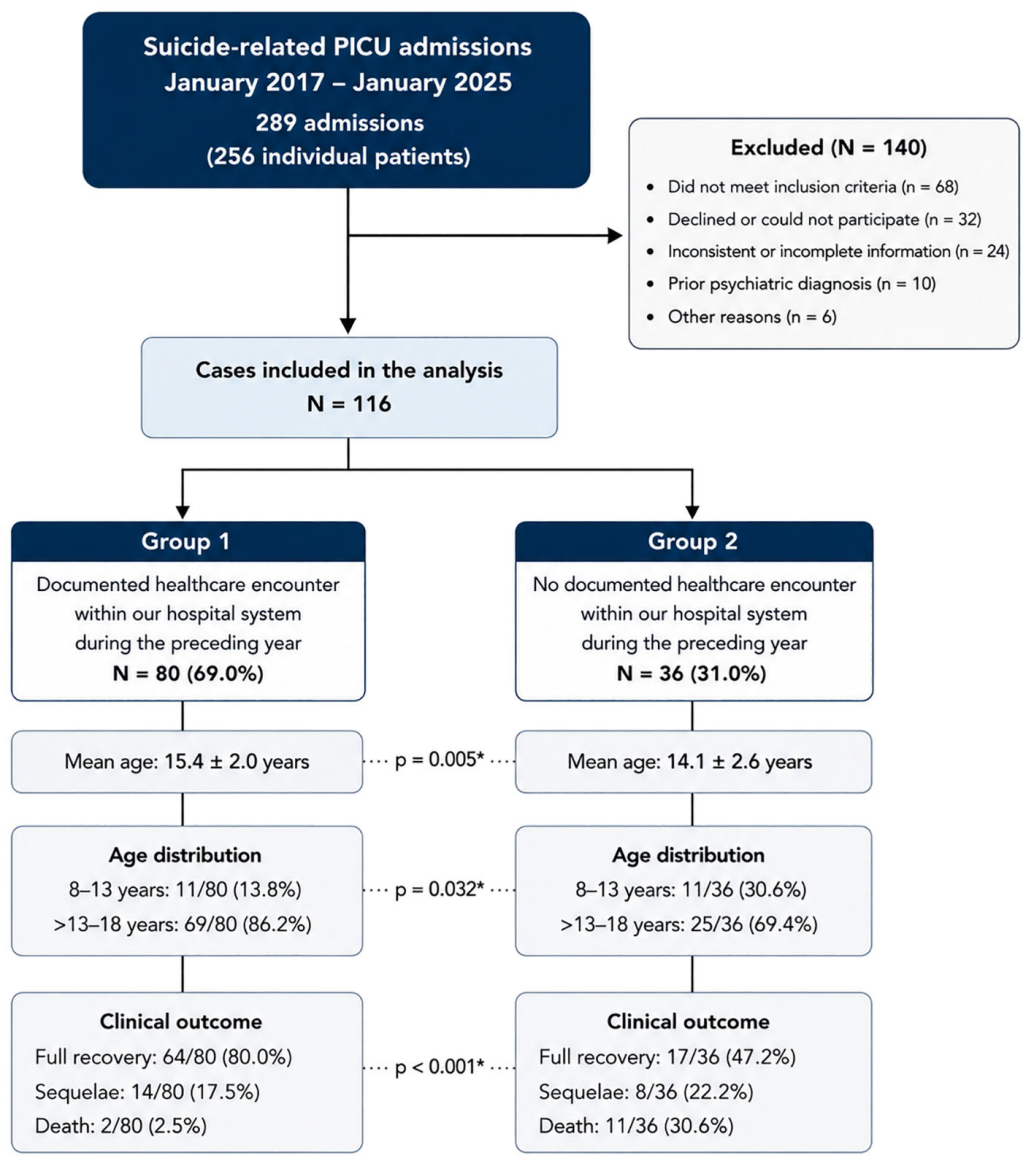
Flow diagram of study inclusion and comparison of clinical outcomes between patients with documented healthcare encounters during the preceding year (Group 1) and those without documented healthcare encounters within the hospital system during the same period (Group 2).

During the study period, 289 suicide-related PICU admissions involving 256 individual patients were recorded. Following application of the predefined inclusion and exclusion criteria, 116 patients were included in the final analysis (**Figure 1**).

The study cohort consisted of 81 girls (69.8%) and 35 boys (30.2%), with a mean age of 15.5 ± 2.1 years (range, 8–18 years).

Based on the method of suicide attempt, patients were categorized into two groups: self-poisoning and trauma-related attempts.

Among the 98 patients (84.5%) who attempted suicide through self-poisoning, the most commonly ingested substances were central nervous system medications (34%), analgesic or antipyretic agents (31%), rodenticides (20%), antihypertensive, anticoagulant, and antidiabetic medications (12%), and miscellaneous agents such as vitamins, antiemetics, and thyroid medications (3%). Two patients (1.7%) administered medications via intramuscular injection.

Among the 18 patients (15.5%) who used trauma-related methods, hanging was the most common mechanism (61.1%), followed by jumping from a height (22.3%) and firearm-related injuries (16.6%).

### Clinical outcomes

Overall, 22 patients (19.0%) survived with long-term sequelae, whereas 13 patients (11.2%) died following the suicide attempt.

Documented sequelae included cerebral palsy, chronic kidney disease, liver failure, septic shock, orthopedic disabilities, spinal cord injuries, loss of ambulation, amputations, colostomy, uterine rupture or atony, endometrial injury, episiotomy-related complications, rectal injuries, hemorrhagic complications, hypertension, and cardiac arrhythmias.

Fifty patients (43.1%) required advanced PICU interventions. These included endotracheal intubation in 46 patients (39.7%), vasoactive or inotropic support in 19 (16.4%), renal replacement therapy in 8 (6.9%), and extracorporeal membrane oxygenation (ECMO) in 3 patients (2.6%).

The most common indications for PICU admission included respiratory or cardiac arrest, respiratory failure, severe poisoning, altered consciousness, seizures, cardiovascular instability, hypotension, arrhythmias, and indications for dialysis.

### Comparison of patients with and without prior healthcare contact

Patients were stratified into two groups according to the presence of documented healthcare encounters within our hospital system during the year preceding the suicide attempt.

Group 1 consisted of patients who had at least one healthcare encounter during the preceding year for symptoms or presentations subsequently classified as potential warning signs (n = 80, 69.0%).

Group 2 consisted of patients with no documented healthcare encounters within our hospital system during the year preceding the suicide attempt (n = 36, 31.0%).

Because records from external healthcare institutions were not available, healthcare encounters occurring outside our hospital network could not be systematically assessed.

### Healthcare encounters and potential warning signs

Medical records, emergency department visits, outpatient clinic encounters, and interview data were reviewed to identify potential warning signs during the year preceding the suicide attempt. Categories were assigned retrospectively by consensus among the investigators according to the primary reason for presentation. Patients could be assigned to more than one category when multiple warning signs were identified.

Among the 80 patients in Group 1, five major categories of healthcare encounters were identified.

#### Violence-related presentations (n = 30)

Forty-two patients had a documented history of physical violence, and 30 presented to the emergency department with injuries requiring forensic documentation. These encounters involved domestic violence, interpersonal violence, street fights, or assaults involving family members or peers. Three male patients had documented legal convictions related to violent offenses.

Primary point of contact: Emergency Department.

#### Humiliation-related presentations (n = 29)

These presentations were associated with experiences of humiliation related to appearance, body image concerns, academic difficulties, or perceived social inadequacy. Several patients underwent specialist evaluations, laboratory investigations, or educational assessments following concerns raised by family members or teachers.

Primary points of contact: Endocrinology, plastic surgery, orthopedics, and general pediatrics.

#### Recurrent psychosomatic presentations (n = 24)

Patients in this category presented repeatedly with physical complaints for which no clear organic explanation was identified despite repeated medical evaluations. The median number of presentations was six per patient (range, 3–9).

Reported symptoms included chest pain with repeatedly normal cardiac investigations, musculoskeletal pain, abdominal pain, reflux-related complaints, dizziness, syncope, hair loss, and nonspecific somatic symptoms.

Primary points of contact: General pediatrics, physical medicine and rehabilitation, cardiology, gastroenterology, pulmonology, and otolaryngology.

#### Premature discharge against medical advice (n = 27)

These encounters involved patients who were removed from medical care by family members before completion of treatment or who left healthcare facilities without authorization. Underlying admission diagnoses included trauma, poisoning, infection, and gynecological conditions. Formal documentation or police notification was present in all cases.

Primary points of contact: Emergency Department and pediatric inpatient wards.

#### Gynecology-related presentations (n = 43)

Female adolescents presented with gynecological concerns including vaginitis, suspected sexually transmitted infections, pregnancy evaluation, antenatal follow-up, recurrent urinary tract infections, and genital herpes.

Primary points of contact: Gynecology clinics, Emergency Department, and Dermatology.

Because individual patients could be assigned to more than one category, the total number of healthcare encounters exceeded the number of patients.

### Distribution of potential warning signs

The distribution of potential warning signs among patients with prior healthcare contact is summarized in **Table 1**.

**Table 1:** Distribution of warning signs among patients with prior hospital contact (group 1, n = 80).

Primary reason for hospital visit	Total visits	Patients (n/80)
A – Violence	30	30 (37.5%)
B – Humiliation	29	29 (36.3%)
C – Psychosomatic Complaints	24	24 (30.0%)
D – Premature Discharge	27	23 (33.8%)
E – Gynecology*	43	43/58** (74.1%)

*Gynecology visits were only analyzed among female patients.

**Percentage calculated based on the number of female patients (n = 58).

***Patients could present with more than one complaint; therefore, the total number of visits exceeded the cohort size.

### Comparison of clinical outcomes between groups

Patients in Group 1 were older than those in Group 2 (15.4 ± 2.0 vs. 14.1 ± 2.6 years, p = 0.005).

Children aged 8–13 years were more frequently represented in Group 2 than in Group 1 (30.6% vs. 13.8%, p = 0.032).

Clinical outcomes differed significantly between groups. Patients in Group 2 experienced higher rates of mortality and long-term sequelae compared with those in Group 1 (p < 0.001) (**Figure 1**).

The method of suicide attempt differed significantly between groups (**Table 2**). Trauma-related methods were substantially more common among patients without prior documented healthcare contact (Group 2) than among those with prior healthcare contact (44.4% vs. 2.5%, p < 0.001). In contrast, self-poisoning was the predominant method in Group 1 (97.5% vs. 55.6%, p < 0.001).

**Table 2:** Comparison of patients with and without prior hospital visits.

Variable	Group 1 (n = 80, 69%)	Group 2 (n = 36, 31%)	p-value
Age, years (mean ± SD)	15.4 ± 2.0	14.1 ± 2.6	**0.005** ^1^
Age groups			
8–13 years	13.8% (11/80)	30.6% (11/36)	**0.032^2^**
>13–18 years	86.2% (69/80)	69.4% (25/36)	
Sex	0.350		
Female	72.5% (58/80)	63.9% (23/36)	
Male	27.5% (22/80)	36.1% (13/36)	
Self-poisoning	97.5% (78/80)	55.6% (20/36)	<0.001^2^
• Drug ingestion	86.7% (71/80)	50.0% (18/36)	
• Caustic substances (rat poison, detergent)	8.8% (7/80)	5.6% (2/36)	
Trauma-related attempts	2.5% (2/80)	44.4% (16/36)	<0.001^2^
• Hanging	100% (2/2)	56.3% (9/16)	
• Firearms	0% (0/80)	18.8% (3/16)	
• Jumping from height	0% (0/80)	25.0% (4/16)	
Outcome	<0.001*		
Full recovery	80.0% (64/80)	47.2% (17/36)	
Sequelae	17.5% (14/80)	22.2% (8/36)	
Death	2.5% (2/80)	30.6% (11/36)	

^1^ Independent samples t-test, ^2^Pearson chi-square test. Significant p values are shown in bold.

### Association between age, suicide method, and mortality

When survivors (n = 103) were compared with nonsurvivors (n = 13), mean age did not differ significantly between groups (15.0 ± 2.1 vs. 14.5 ± 3.0 years, p = 0.522). Likewise, mortality rates were not significantly different between children aged 8–13 years and adolescents aged 13–18 years (p = 0.062).

In contrast, suicide method was strongly associated with mortality. Trauma-related suicide attempts were substantially more frequent among nonsurvivors than survivors (84.6% vs. 6.8%, p < 0.001), whereas self-poisoning was more common among survivors (93.2% vs. 15.4%, p < 0.001) (**Table 3**).

**Table 3:** Association of age and suicide method with mortality.

Variable	Survivors (n = 103)	Non-survivors (n = 13)	p-value
Mean age (years)	15.0 ± 2.1	14.5 ± 3.0	0.5221
Age groups			
8–13 years	22.3% (23/103)	46.2% (6/13)	0.0622
>13–18 years	77.7% (80/103)	53.8% (7/13)	
Suicide method			
Trauma-related	6.8% (7/103)	84.6% (11/13)	<0.0012
Self-poisoning	93.2% (96/103)	15.4% (2/13)	

^1^ Independent samples t-test, ^2^Pearson chi-square test. Significant p values are shown in bold.

### Mortality according to age and sex

Mortality rates did not differ between sexes in adolescents aged 13–18 years (females 8.1% vs. males 8.0%, p = 0.992). However, among children aged 8–13 years, mortality was significantly higher in boys than in girls (50.0% vs. 5.3%, p = 0.005). Overall mortality was higher among males than females (20.0% vs. 7.4%, p = 0.048). Among children aged 8–13 years, mortality was significantly higher in boys than in girls (50.0% vs. 5.3%, p = 0.005). No sex-related difference in mortality was observed among adolescents aged 13–18 years (8.0% vs. 8.1%, p = 0.992) (**Table 4**).

**Table 4:** Mortality by age group and sex.

Group	Mortality rate	p-value
All children (n = 116)		**0.048** ^1^
Female (n = 81)	7.4% (6/81)	
Male (n = 35)	20.0% (7/35)	
Children aged 8–13 years (n = 29)		**0.005** ^1^
Female (n = 19)	5.3% (1/19)	
Male (n = 10)	50.0% (5/10)	
Adolescents aged 13–18 years (n = 87)		0.992^1^
Female (n = 62)	8.1% (5/62)	
Male (n = 25)	8.0% (2/25)	

^1^ Pearson chi-square test. Significant p values are shown in bold.

### Psychosocial factors identified through patient and family interviews

Based on face-to-face interviews with surviving patients and discussions with family members of deceased children, the following psychosocial factors potentially associated with suicide attempts were identified (**Table 5**). Their associations with sequelae and mortality, stratified by sociocultural and demographic characteristics, are detailed in **Tables 6**, **7**.

**Table 5:** Distribution of psychosocial factors identified through interviews.

Associated factor	n	%
Sexual consent violation	32	27.6
Violence	30	25.9
Humiliation	20	17.2
Pressure	12	10.3
Separation	5	4.3
Child labor	4	3.4
Partner problems	4	3.4
Gender identity related distress	3	2.6
No recognized warning signs	3	2.6
Media influence	2	1.7
Witnessing a relative’s suicide	1	0.9

**Table 6:** Comparison of survivors and non-survivors according to identified associated factors.

Parameter	All patients (n=116)	Survivors (n=103)	Nonsurvivors (n=13)	P value
Violation of sexual consent	27.6% (32/116)	27.2% (28/103)	30.8% (4/13)	0.785
Non-pregnant	75.0% (24/32)	82.1% (23/28)	25.0% (1/4)	0.014*
Pregnant	25.0% (8/32)	17.9% (5/28)	75.0% (3/4)	
Physical violence	25.9% (30/116)	28.2% (29/103)	7.7% (1/13)	0.099
Humiliation	17.2% (20/116)	17.5% (18/103)	15.4% (2/13)	0.605
Pressure	10.3% (12/116)	11.7% (12/103)	0.0% (0/13)	0.194
Separation	4.3% (5/116)	4.9% (5/103)	0.0% (0/13)	Not tested due to small sample size
Gender identity related distress	2.6% (3/116)	0.0% (0/103)	23.1% (3/13)	Not tested due to small sample size
No recognized warning signs	2.6% (3/116)	1.0% (1/103)	15.4% (2/13)	Not tested due to small sample size
Child labor	3.4% (4/116)	3.9% (4/103)	0.0% (0/13)	Not tested due to small sample size
Media influence	1.7% (2/116)	1.9% (2/103)	0.0% (0/13)	Not tested due to small sample size
Partner problems	3.4% (4/116)	3.9% (4/103)	0.0% (0/13)	Not tested due to small sample size
Witnessing a relative’s suicide	0.9% (1/116)	0.0% (0/103)	7.7% (1/13)	Not tested due to small sample size

**Table 7:** Comparison of survivors by discharge status (intact vs. with permanent sequelae).

Parameter	All survivors (n=103)	Intact at discharge (n=81)	Permanent sequela (n=22)	P value
Violation of sexual consent	27.2% (28/103)	28.4% (23/81)	22.7% (5/22)	0.596
Non-pregnant	82.1% (23/28)	91.3% (21/23)	40.0% (2/5)	0.007*
Pregnant	17.9% (5/28)	8.7% (2/23)	60.0% (3/5)	
Physical violence	28.2% (29/103)	30.9% (25/81)	18.2% (4/22)	0.241
Humiliation	17.5% (18/103)	16.0% (13/81)	22.7% (5/22)	0.465
Pressure	11.7% (12/103)	13.6% (11/81)	4.5% (1/22)	0.241
Separation	4.9% (5/103)	6.2% (5/81)	0.0% (0/22)	Not tested due to small sample size
‘No recognized warning signs’	1.0% (1/103)	0.0% (0/81)	4.5% (1/22)	Not tested due to small sample size
Child labor	3.9% (4/103)	2.5% (2/81)	9.1% (2/22)	Not tested due to small sample size
Media influence	1.9% (2/103)	0.0% (0/81)	9.1% (2/22)	Not tested due to small sample size
Partner problems	3.9% (4/103)	2.5% (2/81)	9.1% (2/22)	Not tested due to small sample size

#### Violation of sexual consent (n = 32/116)

Among 44 girls who were married before the age of 18 years, 32 identified circumstances related to non-consensual or coerced marriage as a major factor preceding the suicide attempt. Reported situations included forced marriage (n = 27), arranged engagement (n = 4), and involvement in commercial sexual exploitation (n = 1).

Follow-up data revealed substantial maternal and neonatal morbidity among affected adolescents, including preterm birth, small-for-gestational-age infants, neonatal asphyxia, prolonged NICU admission, postpartum hemorrhage, uterine rupture, rectal injury, and other obstetric complications.

#### Exposure to violence (n = 30/116)

A total of 42 children had histories of physical violence, and in 26 cases, violence was directly identified as the primary reason for suicide. Perpetrators included family members (parents, siblings, relatives, or spouses), peers, or strangers. Some children were involved in violent street altercations, both as victims and aggressors. Additionally, a subgroup of boys (n = 3) faced legal conviction for violent acts. In total, 26 patients attempted suicide after physical abuse, and 4 following verbal/emotional abuse.

#### Humiliation (n = 20/116)

Children reported humiliation due to physical appearance or medical conditions such as precocious puberty, breast hypertrophy, obesity, prominent ears, short stature, anorexia, hirsutism, acne, fungal infections, facial deformities, or scoliosis. These experiences frequently resulted in bullying or self-deprecation.

Academic failure was another key factor: three patients attempted suicide after underperforming in exams, one due to receiving lower grades than siblings, and one following public humiliation by a teacher. Notably, one child who experienced repeated humiliation attempted suicide by hanging and died.

#### Pressure (n = 12/116)

Children described multiple sources of pressure: academic stress (n = 4), parental pressure (n = 6), and peer pressure (n = 3). Although early marriage and child labor were also forms of coercion, they were analyzed separately.

#### Separation (n = 5/116)

Five patients attempted suicide due to separation-related distress: one child living in a dormitory, two abandoned by fathers following remarriage, and two residing in orphanages.

#### Gender identity related distress (n = 3/116)

Gender identity-related distress was identified in three patients. This category was assigned when family members consistently reported persistent distress related to gender expression or perceived incongruence with culturally expected gender roles. No formal psychiatric assessment of gender dysphoria was available. All three patients died following the suicide attempt and had no documented healthcare encounters during the preceding year.

#### Child labor (n = 4/116)

Thirteen children in the study cohort were engaged in child labor. Four attempted suicide: two following intra-family conflict and violence, and two due to economic hardship and the burden of financially supporting their families.

#### Media influence (n = 2/116)

Two patients attempted suicide under the influence of films and social media content.

#### Partner problems (n = 4/116)

Relationship conflicts were identified as triggers in four cases.

#### Witnessing a relative’s suicide (n = 1/116)

One 13-year-old child attempted suicide by hanging after witnessing an older sibling’s suicide. The father reported that the child often expressed missing the sibling deeply.

#### “Children without recognized warning signs” (n = 3/116)

Three patients were described by parents and teachers as academically successful, socially well-adjusted, and without recognized psychosocial stressors or prior healthcare encounters. Two died following the suicide attempt, and one survived with severe neurological sequelae. All belonged to Group 2.

### Additional observations

#### Self-harm (n = 6)

These children engaged in self-injurious behaviors, such as cutting, biting off fingers, or striking their heads and faces. Despite visible injuries, they never presented to hospitals for self-harm. Instead, their hospital visits were linked to unrelated complaints such as acne, short stature, reflux, or chest pain.

#### Right to education (n = 61)

All girls married under 18, those forced into marriage, and all identified child laborers had discontinued school (100%). Additional cases of school dropout were associated with humiliation, violence, or family pressure, though precise percentages could not be determined.

### Psychosocial factors associated with suicide attempts and their association with outcomes

The most frequently identified psychosocial factors associated with suicide attempts in our cohort were sexual consent violation, physical violence, and humiliation. When outcomes were compared across different risk factors, significant differences emerged in terms of mortality (**Table 6**) and long-term sequelae (**Table 7**).

Mortality differed significantly according to several psychosocial factors (**Table 6**). However, findings related to gender identity-related distress (n = 3), children without recognized warning signs (n = 3), and witnessing a relative’s suicide (n = 1) were based on very small numbers and should be interpreted cautiously.

Among survivors, permanent sequelae were more frequent among pregnant adolescents within the violation-of-sexual-consent subgroup and among patients whose suicide attempts were associated with media influence. However, these findings were based on small subgroup sizes and should be interpreted cautiously (**Table 7**).

Because trauma-related methods were substantially more common in Group 2 and were associated with markedly higher mortality, the observed mortality differences between Groups 1 and 2 should be interpreted cautiously.

## Discussion

This study examined children and adolescents admitted to a pediatric intensive care unit following severe suicide attempts and explored potential clinical warning signs and psychosocial circumstances identified during the year preceding the event. Several findings merit attention. First, nearly seven out of ten patients had at least one documented healthcare encounter before the suicide attempt, suggesting that opportunities for earlier recognition may exist within routine clinical practice. Second, children without prior documented healthcare contact were younger, more frequently used trauma-related methods, and experienced substantially higher mortality and morbidity. Third, recurrent psychosomatic presentations, exposure to violence, humiliation-related presentations, and gynecological consultations were common among patients who had interacted with healthcare services before their suicide attempt. Finally, several sociocultural factors—including early or forced marriage, violence, humiliation, school disengagement, and gender identity-related distress—emerged repeatedly during patient and family interviews (1, 2, 12, 13).

Suicidal behavior is increasingly conceptualized as a continuum extending from suicidal ideation to planning, attempts, and completed suicide (14). Children requiring PICU admission represent a particularly severe end of this spectrum because their attempts are often associated with life-threatening injury or poisoning requiring advanced organ support (2, 5, 15). Consequently, studying this subgroup may provide valuable insights into factors that precede the most serious forms of suicidal behavior.

One of the most notable findings of our study was that 69% of patients had at least one healthcare encounter during the year preceding the suicide attempt. Previous studies have similarly shown that many young people who die by suicide have contact with healthcare services in the months before their death (16). Although our study design does not allow determination of whether these encounters were causally related to subsequent suicidal behavior, the findings suggest that healthcare settings may represent important opportunities for identifying children experiencing significant psychosocial distress. Importantly, many of the presentations observed in our cohort—including recurrent unexplained somatic complaints, violence-related injuries, humiliation-related concerns, and gynecological consultations—occurred outside psychiatric services and involved specialties not traditionally associated with suicide prevention.

In contrast, patients without documented healthcare encounters during the preceding year experienced significantly worse outcomes. These children were younger, more frequently used highly lethal trauma-related methods, and had substantially higher mortality rates. Because trauma-related methods were strongly associated with death in our cohort, differences in method selection likely contributed to the observed outcome differences between groups. Nevertheless, the findings highlight a potentially vulnerable subgroup of children who may have limited contact with healthcare systems before a severe suicide attempt. Further research is needed to determine whether reduced healthcare utilization itself contributes to risk or simply reflects other underlying social, familial, or psychological factors.

The higher mortality observed among boys aged 8–13 years deserves particular attention. Although age was not independently associated with mortality in our analyses, younger boys were disproportionately represented among fatal cases. Similar trends have been reported internationally, where suicide rates among younger adolescents have increased substantially during the past two decades (1, 2, 17–19). Because our study lacked a non-suicidal comparison group, these findings should not be interpreted as evidence that younger boys are inherently at higher risk for suicide. However, they suggest that severe suicidal behavior can occur even in children who may not fit traditional adolescent risk profiles and may therefore be more difficult to identify in routine clinical practice.

Similar observations have recently been reported in Türkiye. Torun et al. demonstrated significant increases in both suicidal ideation and suicide attempts among adolescents during the COVID-19 pandemic and emphasized the importance of routine psychosocial screening in adolescent healthcare settings (10). These findings support the need for greater vigilance and earlier identification of vulnerable youth during routine medical encounters.

Several psychosocial circumstances repeatedly emerged during interviews. Exposure to violence, humiliation, body image concerns, school-related difficulties, family conflict, and social adversity were frequently reported by patients and relatives. These findings are broadly consistent with previous literature identifying adverse childhood experiences as important correlates of suicidal behavior (1, 5, 6, 20). Although causality cannot be inferred from our data, the recurrence of these themes across multiple cases suggests that clinicians should remain attentive to psychosocial distress even when children present with seemingly unrelated physical complaints.

Particularly noteworthy was the frequency of recurrent psychosomatic presentations. Many patients had sought medical attention repeatedly for symptoms such as chest pain, abdominal pain, dizziness, syncope, musculoskeletal complaints, or other unexplained somatic symptoms before their suicide attempt. Similar observations have been reported in previous studies demonstrating associations between emotional distress and recurrent healthcare utilization (16). While psychosomatic symptoms are common and nonspecific, repeated presentations without a clear organic explanation may provide an opportunity for broader psychosocial assessment.

Another important observation involved girls presenting with gynecological concerns, including pregnancy-related consultations, sexually transmitted infections, and other reproductive health issues. In our cohort, early marriage and circumstances involving violations of sexual consent were frequently reported among female patients. Pregnant adolescents within this subgroup experienced particularly poor outcomes. However, these findings were based on relatively small numbers and should be interpreted cautiously. More broadly, they underscore the importance of considering psychosocial vulnerability when evaluating adolescents presenting with reproductive health concerns, particularly in regions where early marriage remains prevalent.

Gender identity-related distress was identified in three patients, all of whom died following the suicide attempt. Although this observation is consistent with literature describing elevated rates of suicidal behavior among gender-diverse youth (1, 9, 21), the very small number of affected patients precludes meaningful conclusions regarding mortality risk. Nevertheless, the finding highlights the need for supportive environments and timely access to mental health services for young people experiencing gender-related distress.

The findings of this study also emphasize the importance of sociocultural context. The experiences reported by participants were shaped by local cultural norms, family structures, educational opportunities, and socioeconomic conditions. Factors such as early marriage, school dropout, child labor, family violence, and stigma surrounding gender expression may vary substantially across regions and countries. Consequently, suicide prevention strategies should be adapted to local contexts rather than assuming that risk patterns are identical across populations.

From a public health perspective, our findings support multidisciplinary approaches to suicide prevention. Healthcare professionals, educators, social workers, public health authorities, and families all occupy positions from which warning signs may be recognized. Because many of the identified warning signs occurred outside mental health settings, improving awareness among non-psychiatric clinicians may be particularly valuable. School-based and family-based interventions may also play important roles, especially in communities where psychosocial difficulties are less likely to come to clinical attention.

## Strengths and limitations

Several limitations should be considered when interpreting these findings. The retrospective single-center design limits generalizability and precludes causal inference. Information regarding deceased patients relied partly on retrospective family interviews and may therefore be subject to recall and reporting bias. Healthcare encounters outside our hospital network could not be systematically evaluated, potentially resulting in misclassification of some patients as having no prior healthcare contact. The exclusion of patients with established major psychiatric disorders may have introduced selection bias and limits applicability to the broader population of adolescents with suicidal behavior. In addition, several subgroup analyses involved small numbers of patients, reducing statistical precision and increasing the possibility of chance findings. Finally, the absence of a non-suicidal comparison group prevents estimation of the predictive value of the identified warning signs.

Despite these limitations, this study has several strengths. It represents one of the largest PICU-based cohorts of pediatric suicide attempts reported from a socioeconomically disadvantaged region and provides detailed information regarding healthcare utilization, psychosocial circumstances, and clinical outcomes. By focusing on warning signs observable during routine healthcare encounters, the study offers practical observations that may help clinicians recognize vulnerable children and adolescents who could benefit from further psychosocial assessment.

## Conclusion

In this cohort of children and adolescents admitted to a pediatric intensive care unit following severe suicide attempts, nearly 70% had documented healthcare encounters during the preceding year. Recurrent psychosomatic complaints, violence-related presentations, humiliation or bullying-related concerns, and gynecological consultations were among the most frequently identified warning signs. Children without prior documented healthcare contact were younger, more commonly used trauma-related methods, and experienced substantially worse outcomes.

Although the identified warning signs should not be interpreted as predictors of future suicide, they may represent opportunities for earlier recognition of psychosocial distress during routine clinical encounters. Given the retrospective design, absence of a non-suicidal comparison group, and sociocultural specificity of the study population, the findings should be considered exploratory and hypothesis-generating.

Future multicenter studies incorporating appropriate comparison groups and prospective designs are needed to determine whether these warning signs can contribute to earlier identification of vulnerable children and adolescents and inform culturally sensitive suicide prevention strategies.

## Data Availability

The raw data supporting the conclusions of this article will be made available by the authors, without undue reservation.
